# Synthetic Band Structure Engineering of Graphene Using Block Copolymer-Templated Dielectric Superlattices

**DOI:** 10.1021/acsnano.4c14500

**Published:** 2025-03-06

**Authors:** Moeid Jamalzadeh, Zihan Zhang, Zhujun Huang, Miguel Manzo-Perez, Kim Kisslinger, Takashi Taniguchi, Kenji Watanabe, Pilkyung Moon, Gregory S. Doerk, Davood Shahrjerdi

**Affiliations:** † Electrical and Computer Engineering, New York University, Brooklyn, New York 11201, United States; ‡ Center for Functional Nanomaterials, Brookhaven National Laboratory, Upton, New York 11973, United States; § Research Center for Materials Nanoarchitectonics, National Institute for Materials Science, 1-1 Namiki, Tsukuba 305-0044, Japan; ∥ Research Center for Electronic and Optical Materials, National Institute for Materials Science, 1-1 Namiki, Tsukuba 305-0044, Japan; ⊥ Arts and Sciences, NYU Shanghai, Shanghai 200124, China; # NYU-ECNU Institute of Physics at NYU Shanghai, Shanghai 200062, China

**Keywords:** nanopatterning, superlattice potentials, block copolymer, dielectric nanopatterns, metal oxide superlattices

## Abstract

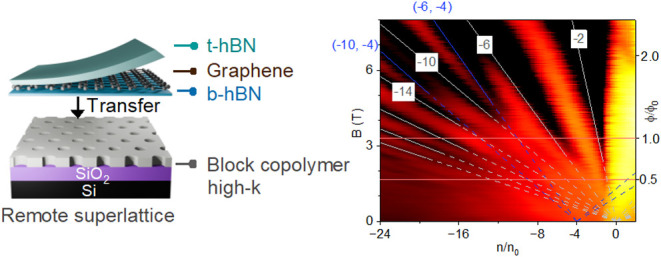

Engineering the electronic band structure of two-dimensional (2D) materials by imposing spatially periodic superlattice (SL) potentials opens a pathway to unconventional electronics. Nanopatterning the gate electrode or surface dielectric near 2D crystals provides a powerful strategy for realizing electrostatically tunable “remote” SLs with flexibility in lattice design. Here, we demonstrate the effectiveness of block copolymer (BCP)-templated dielectric nanopatterns for fabricating etch-free high-grade metal oxide SLs. Alumina (AlO_*x*_) nanopatterns with hexagonal symmetry and a 38 nm SL wavelength are produced as a model material by directly converting a self-assembled BCP film via block-selective vapor phase infiltration. Despite micrometer-scale rotational disorder inherent to BCP self-assembly, electronic transport measurements of graphene reveal replica Dirac points at zero field and Hofstadter mini-gaps under finite magnetic fields. These results indicate the successful formation of remote SL potentials in graphene resulting from optimized AlO_*x*_ nanopattern fabrication to achieve consistent lattice symmetry and periodicity at a macroscopic scale. The findings of this study, combined with the versatile, scalable, and cost-effective nature of BCP nanopatterning, highlight the potential of BCP-templated nanostructures for remote SL engineering in 2D crystals.

## Introduction

Inducing artificially designed and spatially periodic superlattice (SL) potentials in two-dimensional (2D) materials is a powerful strategy for modifying their intrinsic energy band structure. Moiré materials are the archetype of this approach, created by integrating SL potentials into the host layers during fabrication through precise control of lattice mismatch^1−3^ and twist angle^4,5^ between stacked 2D layers. These designer materials have been instrumental in studying exotic correlated phases^6−9^ and exploring unconventional electronics.^10−14^ However, fabricating moiré materials has limitations, such as balancing twist angle relaxation^15−17^ with blister-free heterostructure fabrication.^18−21^ These challenges often limit moiré materials to micrometer-scale dimensions and restrict fabrication throughput.

To address these limitations, the remote SL scheme offers an alternative. This approach involves nanopatterning a “remote” substrate, such as a gate electrode^22−24^ or a surface dielectric near 2D crystals.^25,26^ The spatial modulation of the electrostatic field from the artificially designed remote substrate induces SL potentials in the proximal 2D material. By eliminating the need for SL potential formation during the 2D stacking process, this approach allows for independent optimization of the remote substrate and the 2D heterostructure.

Previous research on remote SL has shown that creating observable mini-Brillouin zone edges in graphene requires an SL wavelength (λ) of a few tens of nanometers,^22,23^ which is achievable with current lithography techniques. Electron-beam lithography (EBL) is commonly used in research settings for this purpose, offering flexibility in designing various SL symmetries with a sub-40 nm wavelength. Alternatively, helium-focused ion beam (FIB) lithography can fabricate remote SLs with lattice constants as small as 16 nm.^27^

Existing methods for remote SL nanofabrication involve a two-step process, where an etching step transfers nanopatterns from a lithographically defined template to the remote substrate. While suitable for small-area patterning in research settings, lithographic techniques for achieving nanoscale features with high precision are prohibitively costly, making them less ideal for nanomanufacturing of remote SL. Additionally, developing an etch process to uniformly transfer dense nanopattern arrays (sub-40 nm pitch) at the wafer scale adds significant complexity. This challenge is further compounded by the limitations of etching, which can restrict the choice of remote substrates. For instance, high-permittivity (high-*k*) metal oxide dielectrics, although technologically appealing for integration into electronic devices reliant on electrostatic fields, remain unrealized in remote substrates, likely due to the difficulty of achieving controlled, uniform, and low-damage etching required for nanopatterning.^28−30^ These challenges underscore the need for an “etch-free” approach to fabricating remote SLs using high-*k* nanopatterns, enabling enhanced functionality and scalability.

In contrast to lithographic techniques, block copolymer (BCP) self-assembly provides an effective, inexpensive, and rapid method for generating periodic nanopatterns over macroscopic dimensions.^31,32^ Like their lithographic counterparts, BCP-based polymeric nanotemplates can produce nanopatterns in other materials through a subsequent etch process. To date, BCP-based nanopatterning has been used to etch 2D materials directly, enabling band structure engineering such as the opening of a band gap in graphene.^33^ However, an unexplored opportunity lies in utilizing self-assembled BCP thin films combined with block-selective vapor phase infiltration (VPI)^34^ to directly create etch-free high-*k* metal oxide nanopatterns that may be used as remote SLs. In this approach, self-assembled BCP thin films serve as a scaffold for selectively incorporating gaseous metal–organic molecules into one block domain via VPI, forming high-*k* metal oxide dielectric nanopatterns such as alumina (AlO*_x_*) upon reaction with an oxidant (e.g., water) and subsequent polymer removal.^35,36^ Despite this potential, current self-assembled BCPs are limited by polycrystalline nanopatterns with small grain sizes (submicrometer) and defect-rich grain boundaries where abrupt grain rotation occurs.^37,38^ These defects disrupt the uniformity and periodicity required to induce superlattice potentials, ultimately limiting the functionality of the BCP-derived dielectric nanopatterns in remote SL applications.

By using BCP-templated AlO*_x_* nanopatterns as a model system, we demonstrate here the successful application of the BCP self-assembly paradigm for implementing remote SLs in graphene. Specifically, we employ polystyrene-*block*-poly(methyl methacrylate) (PS-*b*-PMMA) thin films blended with PS and PMMA homopolymers, which assemble vertical PS cylinders in a PMMA matrix. Importantly, the added homopolymers act as plasticizers, significantly enhancing assembly kinetics and increasing the size of ordered nanopattern grains. By selectively incorporating AlO_*x*_ into the PMMA matrix through VPI, we directly create hexagonally packed arrays of holes in AlO_*x*_ with a sub-40 nm period. This nanopatterned film serves as the back-gate dielectric for a hexagonal boron nitride (hBN)-encapsulated graphene (BGB) device. Electronic transport measurements of the graphene device indicate effective band engineering by the remote SL, illustrated by the formation of replica Dirac points and associated Hofstadter mini-gaps under finite magnetic fields. These results indicate that the AlO_*x*_ nanopatterns provide sufficiently uniform lattice symmetry and periodicity to induce SL effects, despite their polygrained structure. The versatility and scalability of BCP-templated metal oxides could unlock new opportunities for SL engineering in 2D crystals.

## Results

### Remote SL Using Metal Oxide Nanostructures

**Figure 1:**
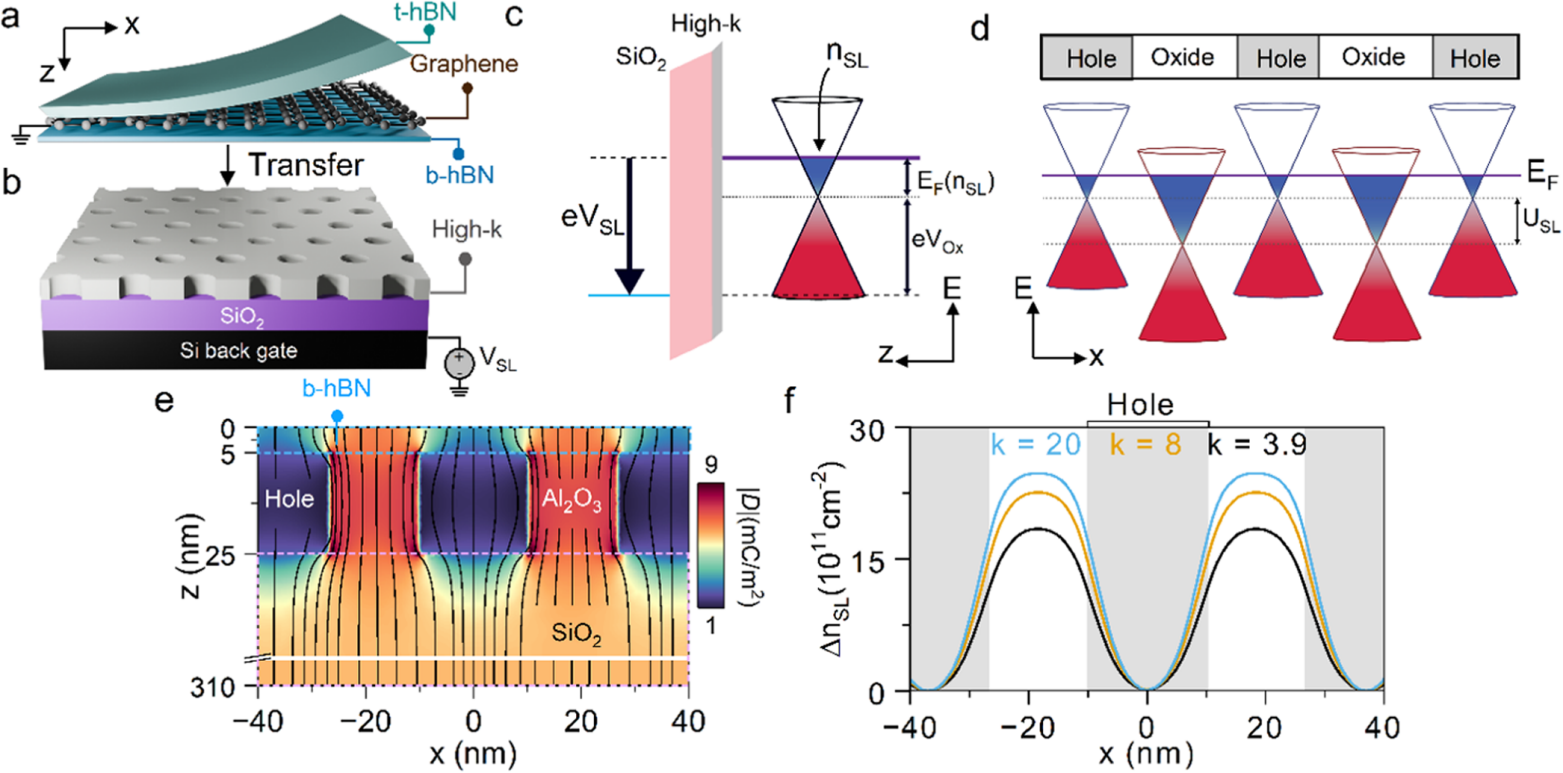
Effect of high-*k* nanopatterns on SL potential. Schematic illustrations of the fabrication process showing (a) the BGB stack transferred onto (b) the remote SL substrate consisting of high-*k* nanopatterns. (c) Band diagram along the *z*-axis showing the electronic system in the nonpatterned regions of the remote substrate under a positive *V*_SL_. *V*_ox_ = *e·n*_SL,ox_/*C*_ox_ from eq 1. (d) Band diagram of graphene along *x*-axis, illustrating an induced difference in the charge neutrality point (CNP) position due to local variations of the dielectric constant in the neighboring hole and solid regions of the high-*k* nanopattern. (e) A model of the electric displacement, *D*, under graphene for a remote substrate with AlO_*x*_ nanopatterns (*k* = 8) at *V*_SL_ = 50 V. The spatial variations of the field lines represent the local variations of the capacitance. (f) Modeled Δ*n*_SL_ under *V*_SL_ = 50 V corresponding to SiO_2_, AlO_*x*_, and HfO_*x*_ nanopatterned dielectrics. The data illustrate the beneficial effect of employing high-*k* nanopatterns in enhancing the electrostatic strength of SL potential.

Figure 1 schematically illustrates the concept behind the surface dielectric nanopatterning approach for creating remote SL potentials in graphene. In this approach, the BGB heterostructure is fabricated independently of the remote substrate by encapsulating graphene between two insulating hBN crystals (denoted as t-hBN and b-hBN in Figure 1a). We employed the van der Waals (vdW) assembly technique combined with high-temperature lamination to produce the BGB heterostructure (see the Methods section). The bottom hBN (b-hBN) thickness was chosen to be sub-5 nm in our experiments. As confirmed by previous remote SL studies,^25^ this thickness range for b-hBN provides both sharp electrostatic boundaries and high carrier mobility in graphene. The integration of the fully fabricated BGB stack through a dry transfer process onto the independently fabricated remote substrate (Figure 1b) completes the fabrication of the graphene SL structure (see the Methods section).

Previous demonstrations of band engineering using remote SLs utilized lithographically defined nanopatterns etched into SiO_2_ dielectrics.^25,26^ Following trends in the microelectronics industry, however, a natural progression of this remote SL scheme involves the use of high-temperature dielectrics in lieu of SiO_2_. In particular, the use of high-*k* dielectrics as a material choice for realizing downscaled dielectrics would be a crucial step for enhancing the electrostatic capability of this remote SL strategy while ensuring low leakage through the dielectric. Here, we expand on these previous SL studies by investigating the use of a remote substrate consisting of a periodic hexagonal array of nanoscale holes in a high-*k* dielectric layer, as depicted in Figure 1b, to realize the SL potentials in graphene.

To illustrate the enhanced electrostatic control afforded by high-*k* dielectrics, it is instructive to examine the electronic band diagram of the system along the *z*-axis, where graphene resides above the solid regions of the nanopatterned oxide layer (Figure 1c). In this band diagram, the applied voltage bias to the silicon back-gate (*V*_SL_) is related to the voltage drop across the dielectric stack and the Fermi energy (*E*_F_) in graphene. This relationship is described by the equation below:
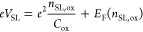
1Here, *n*_SL,ox_ is the electrostatically induced carrier density in this region of graphene, *E*_F_ is the Fermi energy measured from the charge neutrality point (CNP) at *n*_SL,ox_, and *C*_ox_ denotes the equivalent capacitance of the SiO_2_/high-*k* stack shown in Figure 1b. According to this capacitance model, at a given *V*_SL_, increasing the capacitance of the surface dielectric (e.g., by increasing the dielectric permittivity; *k*) leads to a corresponding increase in *n*_SL,ox_. The increase in *n*_SL,ox_ manifests as a larger separation between the *E*_F_ and CNP.

In contrast, the nanopatterned holes in the dielectric layer significantly reduce the gate capacitance in these regions. As a result, the carrier density induced by the *V*_SL_ in graphene above the holes (*n*_SL,h_) is much lower than that *n*_SL,ox_. As shown in Figure 1d, the difference between *n*_SL,ox_ and *n*_SL,h_ creates an energy separation between the CNP of graphene above the solid regions and geometric holes, inducing a periodic SL potential (*U*_SL_) in graphene. This analysis, combined with the capacitance model, suggests that *U*_SL_ can be increased at a fixed *V*_SL_ by increasing the *k* value of the nanopatterned dielectric. Specifically, this modification selectively increases *n*_SL,ox_ while negligibly affecting *n*_SL,h_.

We performed numerical calculations in COMSOL using electrostatic models to illustrate how the permittivity of the nanopatterned dielectric affects the *U*_SL_ strength in graphene at a fixed *V*_SL_. We considered three *k* values: 3.9 for SiO_2_, 8 for AlO_*x*_, and 20 for HfO_*x*_. For simplicity, we used the difference between *n*_SL,ox_ and *n*_SL,h_ (i.e., Δ*n*_SL_ = *n*_SL,ox_ – *n*_SL,h_) as a proxy for *U*_SL_ strength. Figure 1e shows an example of the modeled displacement field (*D*) at *V*_SL_ = 50 V for a remote substrate structure with a nanopattern period of 38 nm, consisting of three dielectric layers: 285 nm unpatterned SiO_2_, 20 nm of AlO_*x*_ nanopatterns, and 5 nm of hBN. The 20 nm thickness for the dielectric nanopatterns was determined through additional simulations to identify its optimal thickness for integration with 285 nm thick SiO_2_ (see Figure S1c). The data in Figure 1e show the spatial variations of *D* in graphene (at *z* = 0) along the *x*-axis above the solid and hole regions of the nanopatterned dielectric. These variations can be linked to the calculations of Δ*n*_SL_, for which we also repeated the simulations for structures with different *k* values (see Note S1). Figure 1g summarizes the results, indicating that the use of high-permittivity nanopatterned dielectrics as remote SLs enhances Δ*n*_SL_, and consequently, *U*_SL_ at a fixed *V*_SL_.

### Fabrication of BCP-Templated AlO_*x*_ Nanopatterns

**Figure 2:**
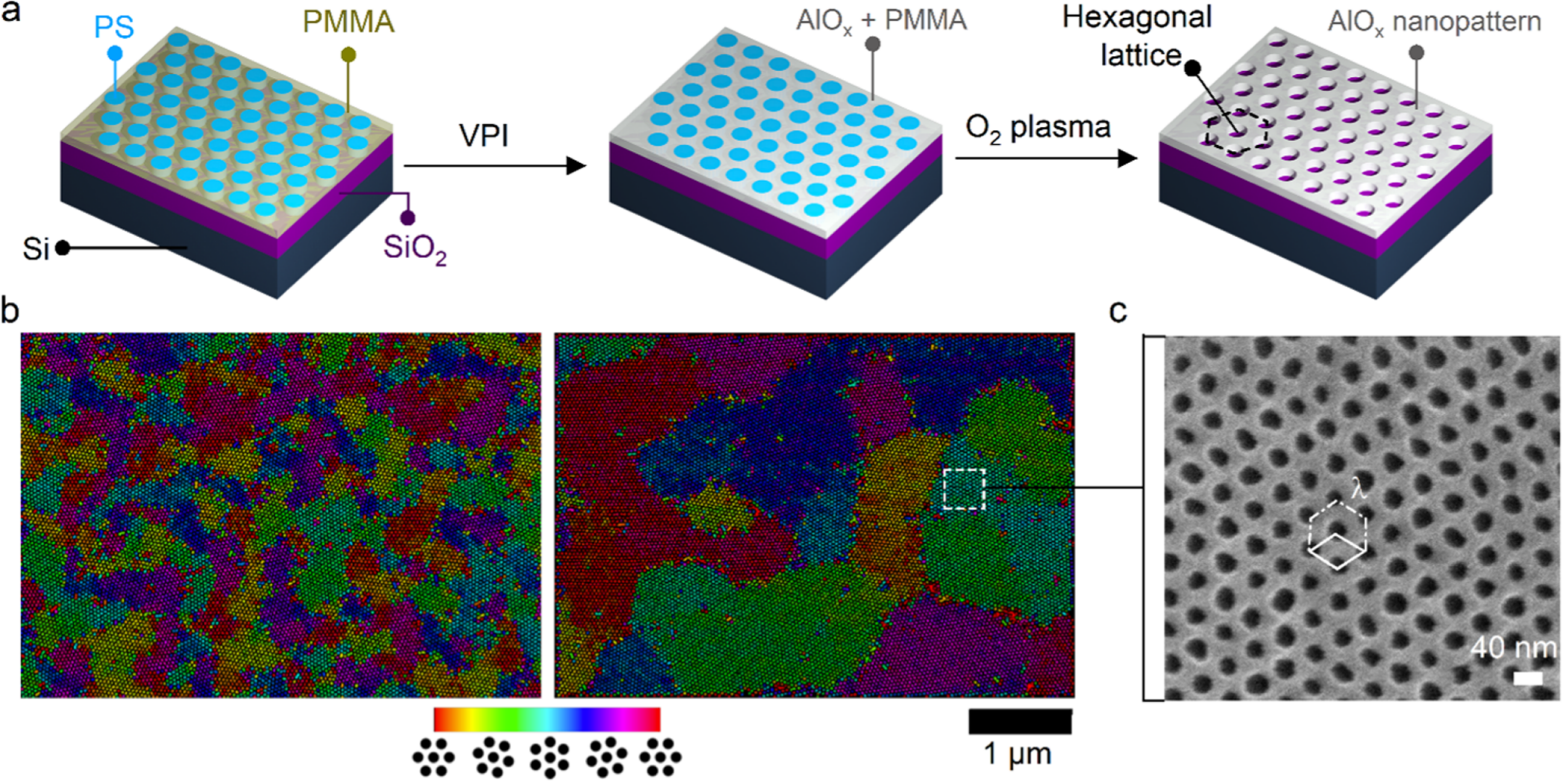
AlO*_x_* nanopattern fabrication. (a) Schematic of AlO_*x*_ nanopattern fabrication, starting with the self-assembly of BCP thin film on SiO_2_ substrate (left), followed by the selective conversion of PMMA block to AlO_*x*_ using VPI (middle), and finally the selective removal of the PS blocks in gentle oxygen plasma (right). (b) Orientation maps of self-assembled hexagonal hole nanopatterns computed from low-magnification scanning electron microscope (SEM) images for the neat PS-*b*-PMMA (left) and the PS-*b*-PMMA/PS/PMMA diblock copolymer/homopolymer blend (right). The scale bar applies to both maps. (c) A high-magnification SEM image from within a grain shows a well-ordered nanopattern with an ∼38 nm hole-to-hole spacing (λ).

Building on the theoretical benefits of high-*k* nanopatterns as remote SLs, we demonstrate the experimental realization of BCP-derived etch-free dielectric nanopatterns with long-range order. These AlO_*x*_ nanopatterns feature a hexagonal lattice and a sub-40 nm period, as shown in Figure 2a and detailed in the Methods section. Previous studies have confirmed that this SL wavelength is sufficient to reveal features like SL-induced replica Dirac cones in transport measurements.^25,39^ Guided by our COMSOL simulations (Figure S1c), we optimized the AlO_*x*_ dielectric nanopattern thickness to 20 nm on a 285 nm SiO_2_ substrate.

A distinct enabling feature of our etch-free BCP-derived AlO_*x*_ nanopattern fabrication process is the self-assembly of vertical PS cylinders with a high degree of pattern order. Although the assembly of well-ordered, vertical PMMA cylinders using asymmetric PS-majority PS-*b*-PMMA across a range of film thicknesses is well documented,^40,41^ there are comparatively fewer reports addressing the opposite case of undirected assembly of PS cylinders in PMMA-majority PS-*b*-PMMA. An early report by Han et al. demonstrated the successful assembly of vertical PS cylinder only in extremely thin films, with a thickness of approximately half the cylinder spacing (∼0.5*L*_0_).^42^ Zhou et al. later demonstrated robust vertical PS cylinder assembly in thicker films by annealing above the temperature in which the PMMA surface energy becomes lower than that of PS (∼270 °C).^43^ Nevertheless, the regions of well-ordered hexagonal lattices of vertical cylinders, or grains, were on the scale of a few 100 nm in films with thicknesses for practical use in nanofabrication (∼1–2*L*_0_), even after 3 h of annealing, implying slow ordering kinetics. The boundaries of these many small grains could introduce substantial disorder in the SL potential for a device on the order of 1–10 μm long due to their tendency to accumulate and stabilize topological defects.^44^

We addressed this issue by blending an asymmetric, PMMA-majority PS-*b*-PMMA BCP with low molecular weight (∼3 kg mol^–1^) PS and PMMA homopolymer plasticizers that significantly accelerate pattern ordering in BCP thin films.^41,45^ Using a blend of a BCP with 40% homopolymer by weight (in a 3:1 PMMA/PS ratio), holey AlO_*x*_ nanopatterns with grain areas more than 1 μm^2^ on average were fabricated from polymer blend thin films annealed for only 5 min at 250 °C. Figure 2b illustrates the color maps of hexagonal lattice orientation comparing films of the neat BCP and the blend described above, both annealed under the same conditions. The data reveal a remarkable increase in grain size afforded by the addition of the homopolymer plasticizers. Specifically, the quantitative image analysis indicates a nominal grain size increase by a factor of 2–3 (see Note S2 for image analysis details).

In Figure 2c, we show an example of a high-magnification scanning electron microscope (SEM) image taken within a grain, indicating excellent uniformity in hole size and pitch (λ). From the low-magnification SEM image of the blend film corresponding to Figure 2b (right), which encompasses multiple grains, real space image analysis yields a mean λ of 38.3 ± 0.1 nm. The same analysis performed on individual grains within the image results in mean λ values tightly clustered around ∼38.2 nm. Analysis performed using fast Fourier transforms agrees well with real space analysis. Thus, while the orientation of the hexagonal lattice rotates between grains, a well-ordered lattice with invariant hole-to-hole spacing is maintained from grain to grain (see Note S2 for image analysis details).

Annealing multiple BCP/homopolymer blend film samples at 250 °C for 5 min indicates a noteworthy degree of sample-to-sample consistency in both the average hexagonal lattice grain sizes and mean λ values (Figures S4 and S5 and Table S2). Hole diameter standard deviations also change little from sample to sample. The mean hole size is larger in these samples, but this depends on the extent of aluminum oxide infiltration, which can be readily tuned by adjusting process conditions such as the number and duration of precursor pulses. The grain sizes of hexagonal hole lattices can be further increased by annealing these BCP/homopolymer blend films for longer times (e.g., 20 min) or higher temperatures (e.g., 260 or 270 °C), as shown in Figure S6 and Table S3. Caution must be taken, however, as the cylinders exhibit a vertical-to-horizontal reorientation with increased annealing time or temperature, which changes λ and ultimately degrades pattern quality (Figure S7); future efforts to co-optimize the selection of the annealing temperature and underlying brush composition may rectify this issue in the future.

The excellent uniformity of the nanopattern periods across rotations of the hexagonal lattice highlights a distinction between the nature of disorder in self-assembled nanopattern remote SLs compared with disorder in moiré SLs. Specifically, moiré SLs frequently exhibit twist angle disorder, manifested as gradual twist angle gradients and sharper transitions.^14^ These variations in twist angle alter the effective moiré SL period, significantly impacting the potential landscape both locally and globally within the device. In contrast, both sharp and gradual rotations of the lattice arise in our self-assembled BCP nanopatterns due to spontaneous nucleation or ordered grains and their gradual coarsening during annealing. While this type of disorder may superficially resemble twist angle disorder in moiré SLs, the competition between enthalpic (phase separation) and entropic (chain stretching) contributions to the system free energy in BCP self-assembly enforces a tightly controlled separation distance between cylinder domains that is maintained except at the high energy grain boundaries. The cylinder spacing is thus effectively unchanged from grain to grain. We hypothesize that this uniform spacing directly translates to high uniformity of the remote SL potential in graphene despite the frequent rotations of the hexagonal lattice. Furthermore, we expect the isotropic band structure of graphene to provide immunity against these rotational disorders, enabling SL-induced band engineering. In the next sections, we discuss experiments that examine synthetic band engineering in graphene using this BCP-templated AlO_*x*_ nanopattern.

### Structure of Graphene SL Device

**Figure 3:**
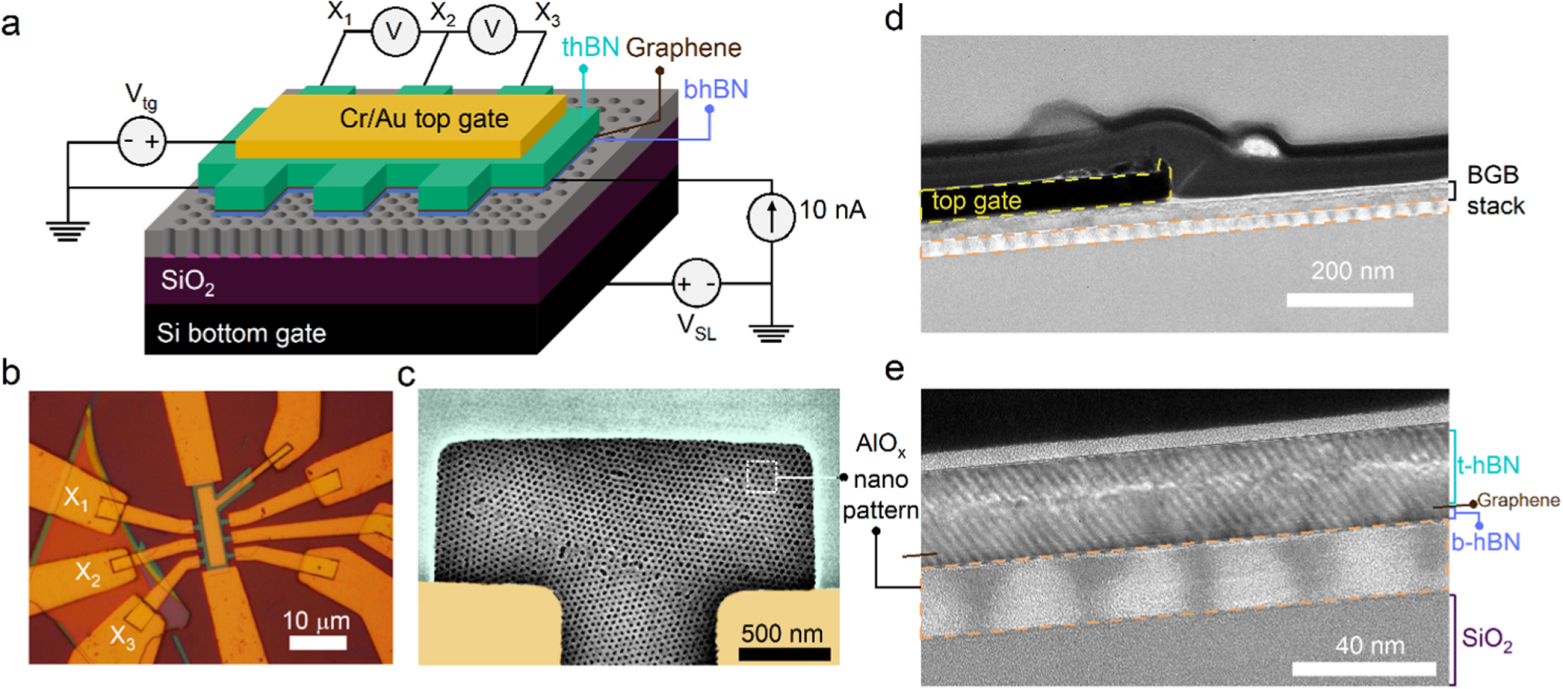
Graphene SL device structure. (a) Schematic illustration of the graphene device in a gated-Hall bar geometry. The back-gate silicon is used for applying *V*_SL_. We used standard lock-in techniques for measuring the electronic transport properties of graphene. (b) Optical image of the fabricated gated-hall bar graphene SL device. (c) High-magnification false-colored SEM image, showing the top-view structure of the device in between two sensing probes. Yellow and green colors denote the Cr/Au metal electrode and the BGB stack, respectively. (d) Transmission electron microscopy (TEM) studies were carried out to further examine the structural properties of the SL device. (e) Close-up TEM image of the structure, indicating the good quality of different interfaces.

Figure 3a shows the schematic illustration of a top-gated graphene SL device in Hall bar geometry. The fabrication process began with the transfer of a prefabricated BGB stack onto the surface dielectric patterned substrate. Notably, the uniformity of the self-assembled AlO_*x*_ nanopatterns across the sample relaxes the placement process of the BGB stack, allowing for flexible positioning on the substrate without the need to align with a predefined site. Subsequently, we employed standard nanofabrication for patterning the active region of the device and forming the different metal electrodes (see the Methods section). Figure 3b shows the optical image of the fully fabricated graphene SL device.

The device width (*W* = 4 μm) and the spacing between the voltage sensing electrodes (*L*_*xx*_ = 3 μm) were intentionally selected to be greater than the grain size of the BCP-templated AlO_*x*_ nanopatterns (∼1 μm^2^). The example scanning electron microscopy (SEM) image in Figure 3c clearly illustrates the presence of rotational disorder in the AlO_*x*_ nanopatterns between two adjacent sensing probes. This experimental design allows us to examine the effect of rotational disorder, inherent in these nanopatterns, on synthetic band engineering of graphene.

We also performed cross-sectional transmission electron microscopy (TEM) to examine the structural properties of the graphene SL device (Figure 3d,e). The TEM study confirmed that the SL graphene device structure employs the target optimal thickness for the BCP-templated AlO_*x*_ dielectric (20 nm) and the b-hBN layer (sub-5 nm). As discussed earlier, these parameters impact the strength of the SL potential in graphene. Moreover, the TEM analysis revealed the atomically sharp interfaces between graphene and hBN layers, essential for achieving high carrier mobility in graphene. Lastly, we observed an apparent variability in hole dimensions (e.g., see Figure 3e). While some variations are expected and observable in SEM analysis (e.g., see Figure 2c), we attribute much of this nonuniformity to differences between the lattice planes and the focused ion beam (FIB) lamella edge. Nonetheless, as we show next, these variations do not disrupt the SL-band engineering in graphene.

### Electronic Transport in Graphene SL

Having established the desired structural properties of the graphene SL device, we then examined how modifying the strength of the SL potential influences the electronic band structure of graphene. To examine these modifications, we performed initial electronic transport measurements at zero field and at a base temperature of 1.5 K using standard lock-in measurement techniques (see the Methods section). As illustrated schematically in Figure 3a, we measured electronic transport in two adjacent graphene regions to investigate the effects of the SL potential and to assess the impact of rotational disorder in the AlO_*x*_ nanopatterns on the band engineering of graphene.

**Figure 4:**
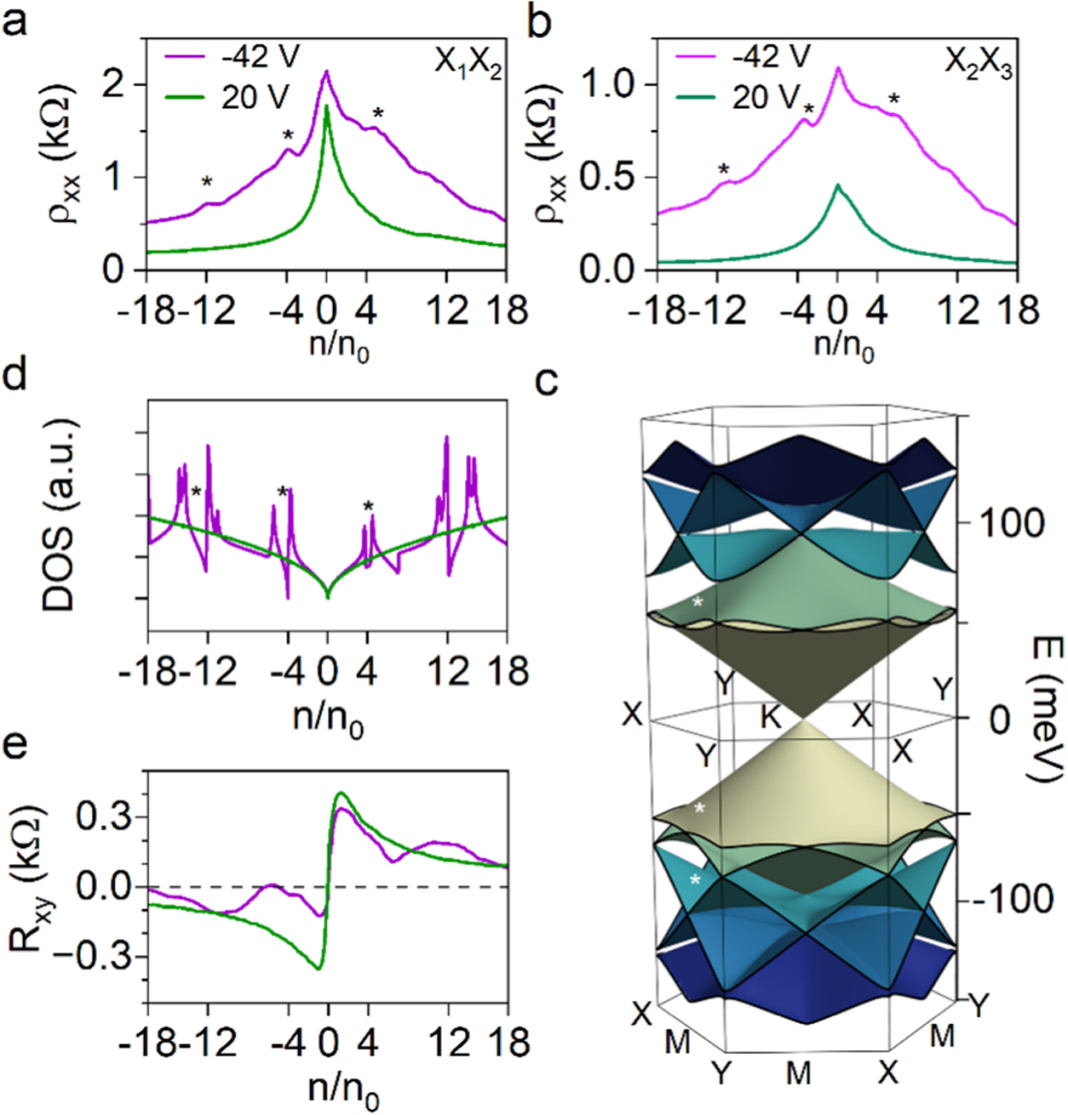
Effect of SL potential on electronic transport. Measured ρ_*xx*_ at zero field plotted against the normalized carrier density under *V*_SL_ of −42 V (purple curve) and 20 V (green curve), corresponding to the (a) *X*_1_*X*_2_ and (b) *X*_2_*X*_3_ regions of the graphene device in Figure 3b. Typical electronic transport in graphene is observed at *V*_SL_ = 20 V, while additional resistance peaks appeared at *V*_SL_ = −42 V. (c) SL-modified graphene electronic band structure modeled at *U*_SL_ = −50 meV, indicating the formation of satellite Dirac cones. (d) Calculated density of electronics states (DOS) in graphene without SL potential (green) and with SL potential (purple). The position of the additional resistance peaks (denoted with “*” symbols) under *V*_SL_ = −42 V in (a) and (b) matches the regions of DOS minimization, indicating the remote SL-induced band engineering in graphene. (e) *R*_*xy*_ data measured at *B* = 200 mT under *V*_SL_ = 20 V (green) and −42 V (purple). The observation of *R*_*xy*_ sign change near *n*/*n*_0_ = −4 under *V*_SL_ = −42 V provides additional evidence for the successful formation of remote SL potential in graphene.

The strength of the SL potential is adjusted by applying a constant voltage bias to the global back gate (*V*_SL_). To probe the effect of the SL potential on electronic properties, we modulated the carrier density in graphene by sweeping the top gate voltage bias (*V*_tg_), while measuring the longitudinal resistivity (ρ_*xx*_) in graphene between the *X*_1_–*X*_2_ and *X*_2_–*X*_3_ sensing probes. In previous studies using surface-patterned SiO_2_ substrates, graphene SL devices showed unmodified transport characteristics at *V*_SL_ = 0 V. In contrast, the graphene SL device in our experiments exhibited typical ρ_*xx*_ behavior (i.e., a single resistance peak located at CNP) when *V*_SL_ was set to 20 V (green curves in Figure 4a,b), suggesting the presence of negative fixed charges in the AlO_*x*_ nanopatterns, a common feature in AlO_*x*_ thin films.^41^ Using a simple capacitance model, we estimated a negative charge density of 5.9 × 10^11^ cm^–2^ in the BCP-templated AlO_*x*_ film (see Note S4).

To understand the expected effect of *V*_SL_, let us first examine the theoretical electronic band structure of graphene. According to the band structure model in Figure 1, increasing the magnitude of *V*_SL_ shifts the CNP away from *E*_F_ more in the dielectric regions than the holes within the dielectric layer. This difference creates a voltage-tunable periodic SL potential, *U*_SL_, within graphene, whose magnitude increases with the *V*_SL_. At sufficiently strong SL potentials, theory predicts that the electronic band structure of graphene is modified, resulting in the formation of replica Dirac cones away from primary Dirac cone. The position of these replica Dirac cones in the electronic band structure depends on the wavelength of the SL potential. Figure 4c shows the modeled band structure of SL-modified graphene assuming an SL wavelength of 38 nm and *U*_SL_ of −50 meV, approximating our system at *V*_SL_ = −42 V. The modeled band structure indicates the asymmetry in the newly generated Dirac cones between the electron and hole regions, with a clear linear crossing and some band overlap in the hole region.

With insights from this theoretical analysis, we next investigated the modification of the electronic properties due to the SL-modified band structure at *V*_SL_ = −42 V. The purple curves in Figure 4a,b show the measured ρ_*xx*_ as a function of the normalized carrier density (*n*/*n*_0_) in graphene between the *X*_1_–*X*_2_ and *X*_2_–*X*_3_ sensing probes, respectively. Here, *n*_0_ represents the fundamental density unit within an SL, calculated from the inverse of the SL unit cell area (*A*). The data reveal satellite resistance peaks at *n*/*n*_0_ = ±4 and −12. The observed modified electronic properties of graphene provide initial evidence for the synthetic band engineering of graphene by the electrostatic remote SL. Moreover, the formation of well-defined resistive peaks in the hole region compared to their counterparts in the electron region appears to be consistent with the modeled SL-modified band structure in Figure 4c. Another crucial observation from this data set is the consistency of the satellite peaks across ρ_*xx*_ measurements from different sensing probes. This observation suggests that rotational disorder in BCP-templated AlO_*x*_ nanopatterns does not disrupt SL-induced band modification, highlighting the robustness of the observed effect.

To better understand the origin of the new features in the transport characteristics of graphene, we examined the theoretical density of electronics states (DOS) at the same SL voltage (Figure 4d). This analysis reveals the modifications of DOS near *n*/*n*_0_ = ±4 and ±12, corresponding to the SL Brillouin zone boundaries. By comparing the transport data with the modeled DOS, we observe an excellent agreement between the position of the satellite resistance peaks and regions of DOS minimization, denoted with “*” symbols. This analysis provides further evidence for band modification in graphene by the electrostatic remote SL.

We attribute this successful band modification of graphene to our optimized process for producing self-assembled BCP-templated AlO_*x*_ nanopatterns with consistent lattice symmetry and periodicity across the entire length scale of the graphene device. Furthermore, and equally impressive, the transport measurements in Figure 4 feature the capability of the AlO_*x*_ nanopatterns to dynamically tune the SL potential in graphene by applying an external voltage to the global back gate of the device.

### Magnetoresistance Measurements of Graphene SL Device

To increase confidence in the assigned origin of the *V*_SL_-tunable features in our transport measurements, we next characterized the electronic properties of graphene under magnetic fields. The modeled band structure in Figure 4c shows the formation of replica Dirac cones with a transition from electron-like to hole-like dispersions. Notably, such a transition manifests as a sign reversal in the transverse resistance (*R*_*xy*_) of graphene at a low magnetic field. In Figure 4e, we show the corresponding *R*_*xy*_ of the graphene device at *B* = 200 mT when applying *V*_SL_ of −42 V (the purple curve) and 20 V (the green curve). The data reveal the sign reversal of *R*_*xy*_ near *n*/*n*_0_ = −4 only at *V*_SL_ of −42 V and not at 20 V. This observation further confirms the electronic band modification of graphene by the AlO_*x*_ nanopattern SL.

**Figure 5:**
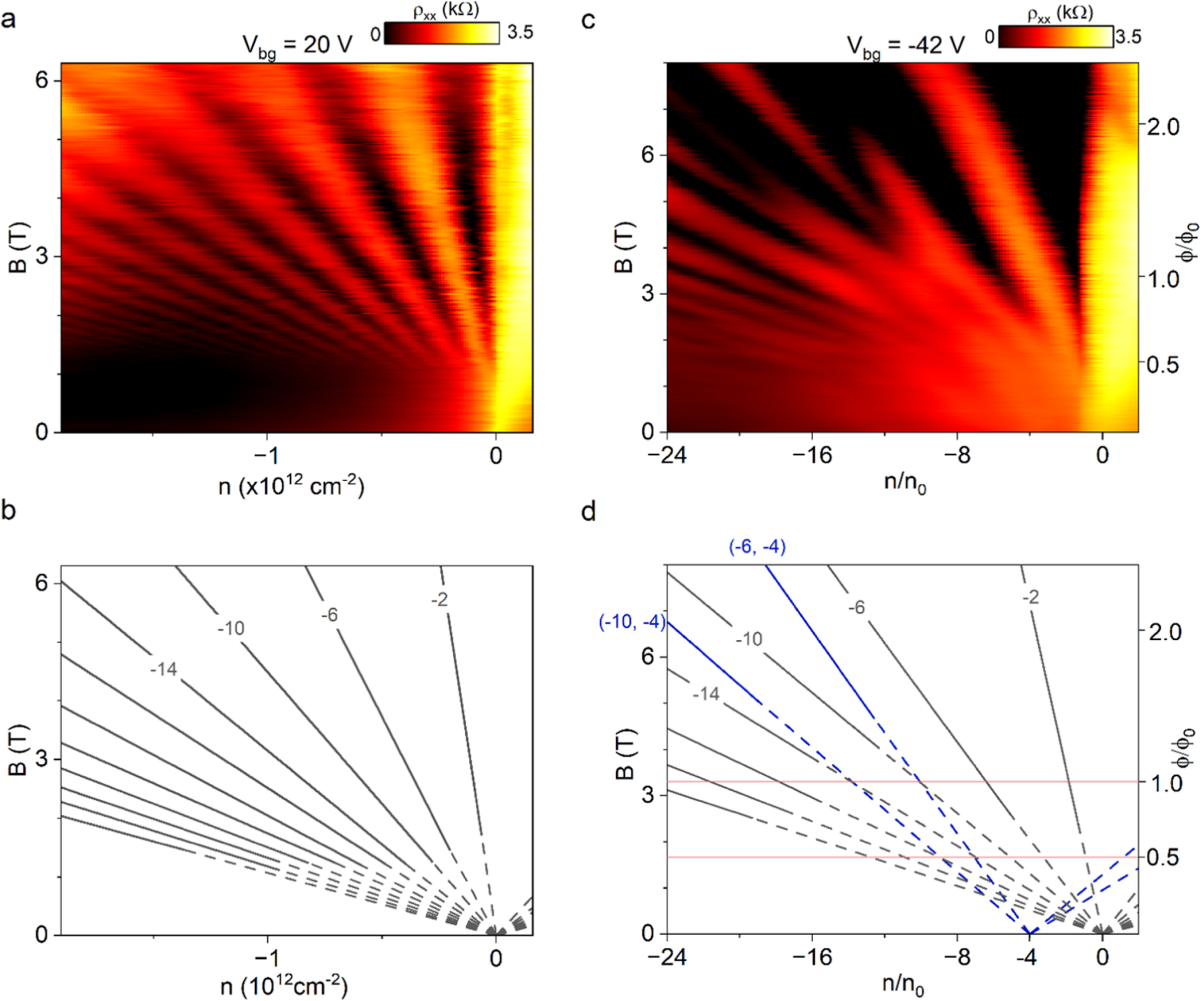
Observation of Hofstadter mini-gaps under magnetic fields. Magnetotransport response of the SL graphene device measured under (a) *V*_SL_ = 20 V and (c) *V*_SL_ = −42 V. The corresponding Wannier diagrams of the magnetotransport response at (b) *V*_SL_ = 20 V and (d) *V*_SL_ = −42 V. The black traces indicate the conventional Landau levels in graphene, while the Hofstadter mini-gaps associated with the satellite peaks of the remote SL are denoted with blue lines. The horizontal red lines show the magnetic field where the conventional Landau levels are expected to intersect with the fan-like Hall states originating from *n*/*n*_0_ = −4.

Having confirmed band modification at low magnetic fields, we explored the magnetoresistance under higher magnetic field conditions to gain deeper insights into the SL-induced quantum effects in graphene. In SL electronic systems, fractal butterfly features emerge under strong magnetic fields arising from the interplay between remote SL and magnetic length scales. In contrast, once the SL potential is deactivated by adjusting *V*_SL_, the graphene electronic system is expected to exhibit only the conventional Landau gaps. Figure 5a illustrates the fan diagram of the graphene device between the *X*_1_–*X*_2_ sensing probes at *V*_SL_ = 20 V, corresponding to the deactivated SL system. These measurements confirm the emergence of only the conventional Landau levels in graphene, as identified by the Wannier diagram in Figure 5b.

To further investigate the effects of the electrostatic SL, we measured the magnetoresistance of graphene at *V*_SL_ = −42 V, where the SL potential is fully activated. In Figure 5c, we plot the corresponding ρ_*xx*_ as a function of the carrier density and applied *B*. In contrast to the deactivated SL state shown in Figure 5a, additional fan-like quantum Hall states emerge, converging at *n*/*n*_0_ = −4, indicating modification of the graphene band structure. To verify whether these new states correspond to the Hofstadter mini-gaps, we mapped the fan diagram using two topological integer numbers (*t*, *s*), satisfying the Diophantine equation:
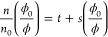
2where ϕ and ϕ_0_ denote the flux per SL unit cell and the magnetic flux quantum, respectively. This analysis confirmed that the new quantum Hall states align with the predicted Hofstadter mini-gaps, as shown in the Wannier diagram in Figure 5d. Collectively, these magnetoresistance measurements confirm the successful band engineering in graphene, driven by the tunable strength of the electrostatic remote SL.

Lastly, the graphene device used in our experiments formed a moiré-patterned SL with a wavelength of ∼10.9 nm due to the rotational alignment between graphene and the hBN insulators. As a result, a pronounced secondary Dirac point was observed at a hole carrier density of 3.8 × 10^12^ cm^–2^, which is distinct from the carrier density corresponding to the mini-Brillouin zone edges created by the electrostatic SL. To maintain focus on the specific features of the electrostatic remote SL, we did not present the full range of data in the earlier figures. The complete data set, which includes the moiré pattern features, is available in Notes S3, S5, and S6. Notably, our data indicate no apparent interactions between the moiré SL and the dielectric-induced electrostatic SL for the range of the *V*_SL_ bias, carrier density, and magnetic field applied in this study.

## Conclusions

The results presented here establish the effectiveness of etch-free BCP-templated dielectric nanopatterns as electrostatic remote SLs for synthetic band engineering in graphene. By incorporating homopolymers as plasticizers, we optimized the BCP self-assembly process, accelerating the assembly kinetics and increasing the size of ordered nanopattern grains. Despite micrometer-scale rotational disorder inherent to self-assembled BCP films, the optimized AlO_*x*_ nanopatterns achieved consistent lattice symmetry and periodicity at macroscopic scales, essential for inducing a robust periodic SL potential. Transport studies of a double-gated graphene test device revealed satellite Dirac points at zero field and Hofstadter mini-gaps under finite magnetic fields, confirming the successful formation of SL potentials consistent with theoretical predictions.

The success of this study in establishing remote SL using self-assembled BCP nanopatterns opens several research directions for further exploring this promising paradigm. Advances in the self-assembly of BCPs have significantly expanded the library of morphologies available for use in this remote SL paradigm.^46^ For instance, while hexagonally packed cylinders are a well-established “native” pattern type readily achievable using coil–coil diblock copolymers, a variety of non-native square, rectangular, and more complex symmetries have been reported through iterative assembly of BCPs.^47,48^ Additionally, infiltration synthesis affords a straightforward approach to integrating materials with even higher dielectric constants, such as TiO_*x*_^49,50^ or HfO_*x*_.^51^ Moreover, while our results demonstrate the robustness of remote SL potentials across micrometer-scale grains with lattice rotations, the full effects of disorder in electrostatic remote SL nanopatterns remain unclear. Finally, BCP self-assembly processes can be readily adapted or directed to adjust the overall level of order and defectivity,^41,52^ or even introduce designer point and line defects.^53,54^ Such tunability could help researchers engineer and study the effects of disorder in remote SLs with much higher control and precision.

## Methods

### AlO_*x*_ Nanopattern Fabrication

A 71 kg mol^–1^ PS-*b*-PMMA (*M*_n_ = 50-*b*-21 kg mol^–1^, polydispersity index (PDI) = 1.07) BCP, PS (*M*_n_ = 3.5 kg mol^–1^, PDI = 1.05), and PMMA (*M*_n_ = 3 kg mol^–1^, PDI = 1.14) were purchased from Polymer Source, Inc. and used as received. These polymers were dissolved in propylene glycol monomethyl ether acetate (PGMEA) at a concentration of 2% by weight. These solutions were then blended by weight in a BCP/PS/PMMA ratio of 60:10:30. A hydroxy-terminated random copolymer of PS and PMMA provided by Dow Chemical Co. (PS-*r*-PMMA–OH; 30% styrene, determined by ^13^C NMR) was diluted to 1% (w/w) in PGMEA.

Silicon wafers with a 285 nm thick dry SiO_2_ layer were first cleaned by oxygen plasma treatment using a March Plasma CS1701 reactive ion etcher for 60 s at 20 W and a pressure of ∼100 mTorr. The substrates were then functionalized by grafting a monolayer, hydroxy-terminated PS-*r*-PMMA copolymer “brush” (30% PS) onto the surface via a dehydration reaction. This process involves spin coating a film at 1500 rpm for 30 s, annealing at 250 °C for 5 min under continuous nitrogen purging, and removing the ungrafted random copolymer by spin-rinsing with neat PGMEA at 3000 rpm for 30 s. A ∼50 nm thick polymer blend film was then spin-cast at 1500 rpm for 30 s and thermally annealed at 250 °C for 5 min under continuous nitrogen purging to facilitate self-assembly. The PMMA matrix morphology was subsequently replicated in AlO_*x*_ using VPI. This was performed using four cycles of exposure to trimethylaluminum and water vapor (200 s each) at 85 °C in a Cambridge Ultratech Savannah S100 atomic layer deposition tool. A majority of the polymer film was then removed by oxygen plasma treatment for 60 s at 100 W and a pressure of ∼100 mTorr. To improve the AlO_*x*_ dielectric properties, the films were annealed in an Ar/O_2_ (90:10) ambient at 500 C for 30 min before BGB stack transfer and device fabrication.

### Device Fabrication and Measurements

The BGB stack was fabricated by encapsulating monolayer graphene in between two hBN insulators using a lamination technique at elevated temperatures.^51^ The fully fabricated BGB stack was then transferred onto the BCP-templated AlO_*x*_ nanopatterns by using an elastomeric stamp. Standard nanofabrication was used for fabricating the double-gated graphene device, involving a combination of electron-beam lithography, reactive ion etching, and electron-beam evaporation.^55,56^ The nanopatterned SL graphene device was wire-bonded with an ultrasonic manual wire-bonder and cooled to 1.5 K in a cryo-free Oxford TeslatronPT system. The transport data were obtained using standard lock-in measurements.

## Data Availability

Data supporting the findings of this manuscript are available from the corresponding author upon reasonable request.

## Supporting Information Available

Additional experimental details, including material characterization, device analysis, and COMSOL simulation results (PDF)
