# Supplementary material for: Synthetic Band Structure Engineering of Graphene Using Block Copolymer-Templated Dielectric Superlattices
Source: ACS Nano. 2025 Mar 6;19(10):9885–95. doi: 10.1021/acsnano.4c14500 (PMC11924326; doi:10.1021/acsnano.4c14500)
Supplement: Supplementary file 1 — nn4c14500_si_001.pdf [file nn4c14500_si_001.pdf]

Supplementary information

Synthetic band structure engineering of graphene using block copolymer templated dielectric superlattices

Moeid Jamalzadeh[1,†], Zihan Zhang[1,†], Zhujun Huang[1], Miguel Manzo Perez[1], Kim Kisslinger[2], Takashi Taniguchi[3], Kenji Watanabe[4], Pilkyung Moon[5,6], Gregory S Doerk[2,*], Davood Shahrjerdi[1,]

[1] Electrical and Computer Engineering, New York University, Brooklyn, NY 11201, USA
[2] Center for Functional Nanomaterials, Brookhaven National Laboratory, Upton, NY 11973, USA
[3] Research Center for Materials Nanoarchitectonics, National Institute for Materials Science, 1-1 Namiki, Tsukuba 305-0044, Japan
[4] Research Center for Electronic and Optical Materials, National Institute for Materials Science, 1-1 Namiki, Tsukuba 305-0044, Japan
[5] Arts and Sciences, NYU Shanghai, Shanghai 200124, China
[6] NYU-ECNU Institute of Physics at NYU Shanghai, Shanghai 200062, China

[†] These authors have contributed equally to this work.
* Corresponding authors: gdoerk@bnl.gov, davood@nyu.edu

Supplementary note S1: COMSOL simulations

The block-selective vapor phase infiltration (VPI) process of block copolymer (BCP) thin films represents a streamlined method for fabricating low-damage nanopatterned high-k dielectrics with high resolution. To explore this potential advantage, we modeled the electrostatic characteristics of the superlattice (SL) structure in COMSOL as a function of the different thicknesses and dielectric constants of the dielectric nanopattern. This approach allowed us to assess the impact of the high-k metal oxide on the carrier density modulation profile, shown in Fig. 1f in the main text.

In our model in Fig. S1a, we set the thickness of the hexagonal boron nitride (hBN) insulator between the graphene and the nanopatterned dielectric to 5 nm. Although employing a thinner hBN insulator can result in sharper SL profiles and stronger modulations (see Fig. S1b), further thinning would degrade the graphene mobility due to increased Coulomb scattering, which is undesirable.

In our simulations, we initially examined the optimal thickness of the high-k nanopattern by using AlO_x as the model system. For the remote structure in Fig. S1a, our results indicate that an optimal thickness of AlO_x is in the range of 10-20 nm (see in Fig. S1c). Reducing the thickness below this range causes electric field leakage, which diminishes the electrostatic effect of the dielectric nanopattern (Fig. S1c).

We calculated the spatial modulation of carrier density in graphene (Δn_{SL}) due to the superlattice bias V_{SL} by ignoring the quantum capacitance and using $\Delta n_{SL} = \Delta C \times V_{SL}/e$, where e is the elementary charge and ΔC represents the spatial variations of the dielectric capacitance due to the nanopattern. To calculate ΔC, we used the modeled electric displacement field D data (see Fig. 1e in the main text) and evaluated the corresponding capacitance at each region using $C = |D|/V_{SL}$. The spatial variations of D, due to the nanopatterned dielectric, results in the periodic spatial variations of the capacitance, ΔC.

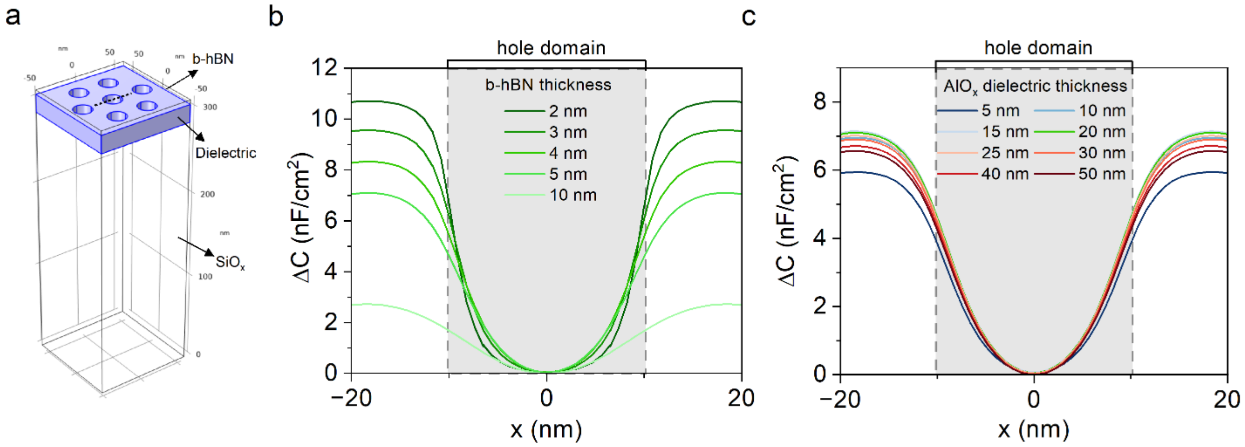

Fig. S1: COMSOL simulations. (a) The schematic illustration of the 3D structure for electrostatic modeling in COMSOL. (b) The modeling results showing the effect of the bottom hBN thickness on the shape profile and amplitude of ΔC. (c) Our simulations provide a guide for the optimal thickness of AlO_x nanopattern in constructing the remote substrate structure of panel (a).

Supplementary note S2: Grain size and superlattice wavelength

S2.a. Grain size analysis

Grain size analysis was done through image analysis using ImageJ. The first step of the analysis involved the Fourier filtering of the raw scanning electron microscope (SEM) images shown in Figs. S2a, S2d to remove low-frequency noise and smooth edges (1 pixel). The contrast was then enhanced, and the images were finally binarized using the default threshold function in ImageJ. The centroid positions of the holes were measured after filtering out smaller features and those on the image edges. The average angle between each centroid and its nearest neighbors was then used to calculate the local orientation of the hexagonal lattice around each hole. Image contrast was inverted, a black background was imposed, and each hole was colored according to the local orientation. In the main text (Fig. 2b), colors were assigned using the "spectrum" look up table such that each orientation is linked with a unique hue.

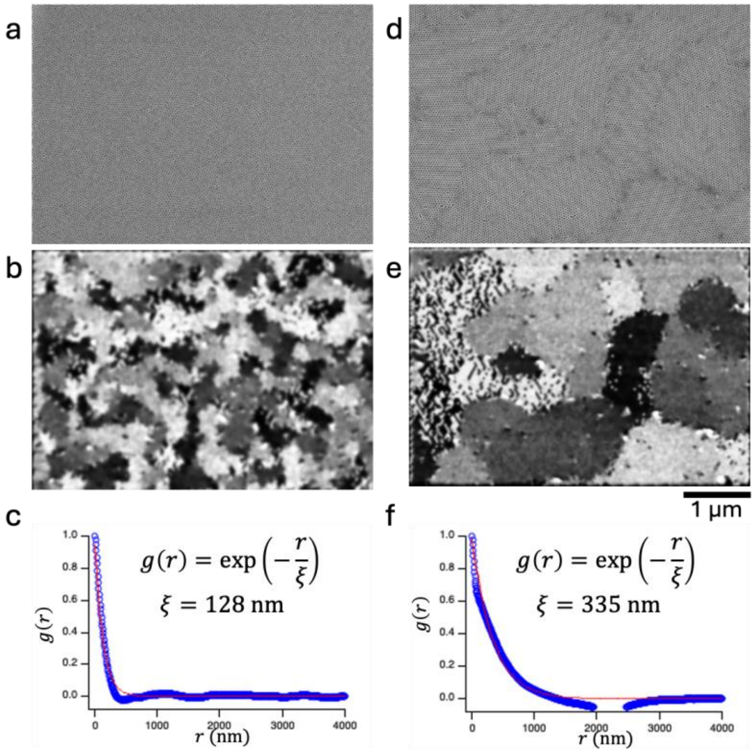

Figure S2: Grain size analysis. (a) Low magnification SEM image, (b) grayscale orientation map, and (c) orientational correlation function plot for the alumina nanopattern fabricated using the neat polystyrene-block-poly(methyl methacrylate) (PS-b-PMMA) block copolymer. (d) Higher magnification SEM image, (e) grayscale orientation map, and (f) orientational correlation function plot for the nanopattern fabricated using the PS-b-PMMA blend with PS and PMMA homopolymers. In both cases, the films were spin-casted, annealed, infiltrated with alumina and etched using an oxygen plasma as described in the text. The orientational correlation decay lengths were obtained using the fit function (red line) provided in (c) and (f). These values underestimate the grain size in comparison to visual inspection, but they do indicate the blend grain size is larger than the grain size for the neat sample by a factor of ~2.6 or more.

To obtain a quantitative measure of the grain size, the holes were shaded in grayscale and a Gaussian blur was used to generate a smoothed orientation map (Fig. S2b, S2e). Next, orientational correlation functions of distance (g(r)) were acquired by computing azimuthally averaged radial intensity profiles. These were calculated from the inverse Fourier transforms of the autocorrelated orientation map Fourier power spectra. These functions were normalized by their initial and final values and were fitted in Igor Pro with an exponential decay function (Fig. S2c, S2f), where the representative decay length (ξ) is taken as a measure of the average grain size. It should be noted that ξ is distinctly smaller than the average grain size (i.e., grain width) apparent by visual inspection of SEM images. This is because the visually apparent grain size is closer to the maximum distance between grain boundaries, while ξ represents an average distance across which the hexagonal lattice orientation decays. Importantly, this decay accounts for both abrupt changes in orientation imposed by grain boundaries and gradual changes within individual grains.

S2.b. Nanopattern hole dimensions

Hole diameters and spacings in the nanopatterned dielectric fabricated using the block copolymer blended with homopolymer were measured via analysis of a low magnification SEM image (Fig. S3a) using ImageJ. The diameter distribution was obtained by real space analysis, while repeat spacings (i.e., pattern period or superlattice wavelength) were acquired through both real space and Fourier space analysis. For real space analysis, images were Fourier filtered to remove low frequency noise and smooth edges (1 pixel). Images were then binarized using the default threshold function and the areas and centroid positions of the holes were measured after filtering out smaller features and holes on the image edges. Individual hole diameters were calculated from hole areas and then averaged. The mean periods (and standard deviations) were calculated from the distances between the centroids of each hole and those of its nearest neighbors. The same analysis was performed in cropped rectangular regions within selected grains, marked by colored dashed lines in Fig. S3a.

Fourier-space analysis was performed by acquiring fast Fourier transform power spectra from the entire image and from within selected grains; exemplary power spectra corresponding to the grains marked by colored dashed lines in Fig. S3a are shown in Fig. S3b. An azimuthally averaged radial intensity profile was computed for each power spectrum, and the first peak was fitted with a Gaussian function in Igor Pro to obtain the peak center position (q_0). The hole period was then calculated as $\frac{4\pi}{q_0\sqrt{3}}$. The uncertainty in the estimated period was obtained from the uncertainty in the fitted peak center position. The real space and Fourier space dimensions calculated by SEM image analysis are provided in Table S1, which indicates excellent agreement between the different calculation methods and a high degree of pattern uniformity and consistency from grain to grain.

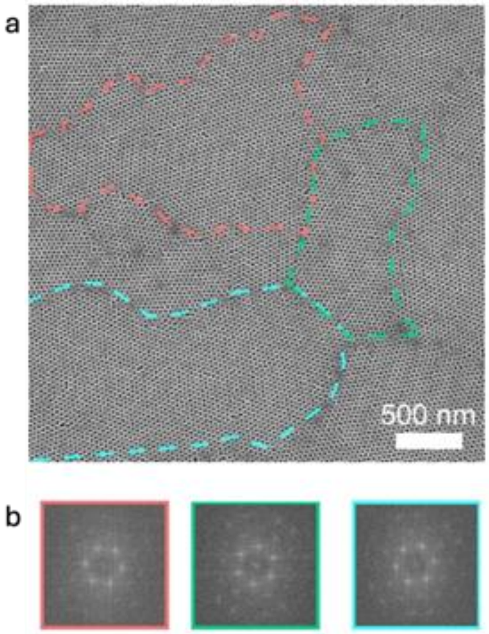

Figure S3: **Fourier-space image analysis** (a) Low magnification SEM image of the nanopatterned dielectric. (b) Fourier power spectra acquired from within grains marked by colored dashed lines. The spectra border colors correspond to the regions in (a) with the same dashed boundary color.

Table S1: Nanopattern Feature Dimensions Obtained through SEM Image Analysis

Region	Real Space [1]				Fourier Space [2]
	Diameter (nm)		Period (nm)		Period (nm)
	Mean[3]	Standard Deviation	Mean[3]	Standard Deviation	Value[4]
Red	21.7 ± 0.2	1.7	38.1 ± 0.2	2.7	38.1 ± 0.3
Green	21.6 ± 0.2	1.9	38.2 ± 0.2	3.0	38.7 ± 0.9
Blue	21.6 ± 0.1	1.6	38.2 ± 0.1	2.7	38.1 ± 0.6
Entire Image	21.6 ± 0.1	2.3	38.3 ± 0.1	3.1	38.1 ± 0.1

[1] The diameter of each hole calculated from the pixel area. Periods were measured from the centroid distances between adjacent holes.

[2] Values were obtained from the center points of the first peak (q_0) in the azimuthally averaged fast Fourier transform power spectrum, acquired through Gaussian fitting. The fitting is given by $\frac{4\pi}{q_0}\sqrt{3}$.

[3] The uncertainty represents 3 times the standard error (99.7% confidence interval).

[4] The uncertainty represents 3 times the estimated standard deviation in the fitted peak center position.

S2.c. Characterization of Nanopattern Sample-to-Sample Variation and the Effects of Varying Annealing Time and Temperature

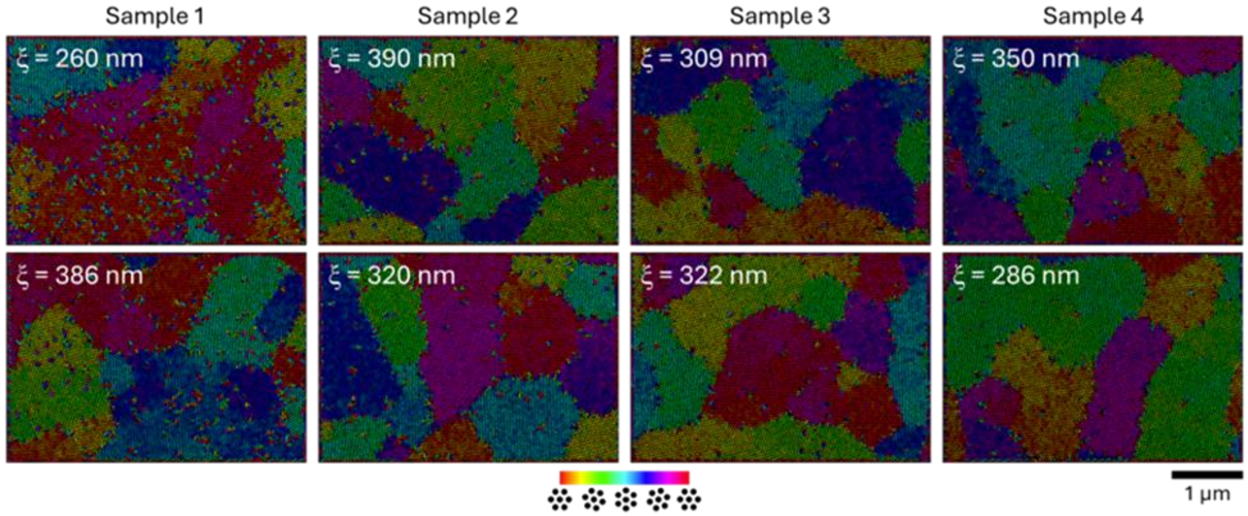

Figure S4: Exemplary orientation maps of self-assembled hexagonal hole patterns computed from low-magnification SEM images for four samples using the PS-b-PMMA/PS/PMMA diblock copolymer/homopolymer blend described in the text. The polymer films were all ~50 nm thick and were annealed for 5 minutes at 250 °C. Samples were infiltrated and etched as described in the text. The corresponding orientational decay length (ξ) at the top of each map.

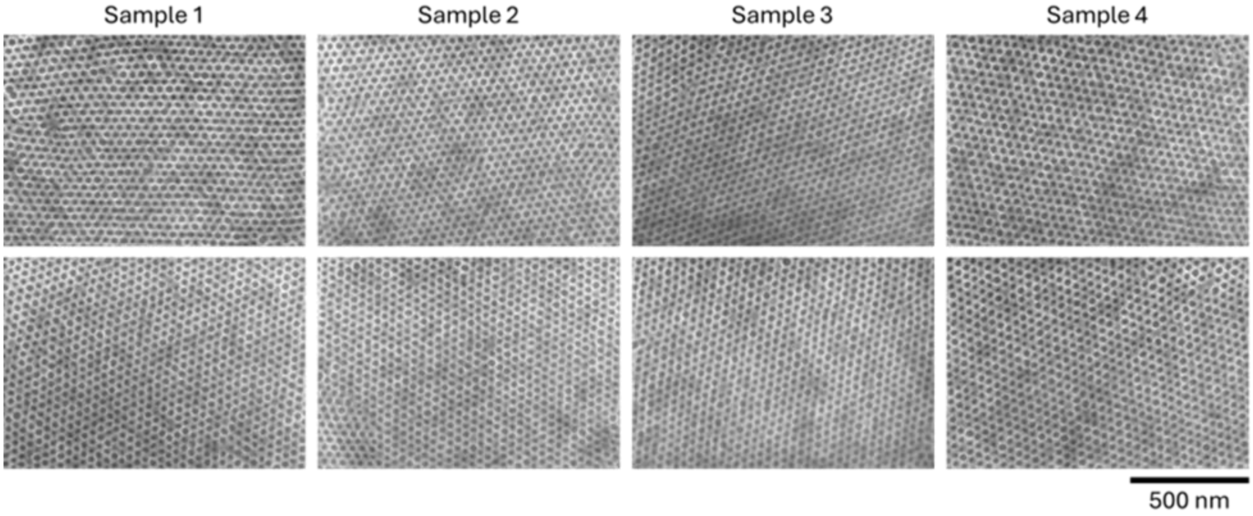

Figure S5: Exemplary high-magnification SEM images acquired from the four samples shown in Figure S4.

Table S2: Image analysis results for 4 ~50 nm thick PS-b-PMMA/PS/PMMA diblock copolymer/homopolymer blend film samples annealed for 5 minutes at 250 °C.

Sample	Image	Period (nm)		Diameter (nm)		ξ (nm)
		Mean	Standard Deviation	Mean	Standard Deviation	
1	1	38.4	3.3	23.2	2.7	260
	2	38.4	3.3	22.9	2.6	386
2	1	38.1	2.9	22.2	2.2	390
	2	38.0	3.0	22.8	2.3	320
3	1	38.2	2.8	22.0	2.0	309
	2	38.3	2.8	22.3	2.0	322
4	1	38.4	2.8	21.8	1.9	350
	2	38.4	2.7	21.7	1.8	286
Average[1]		38.3 ± 0.2	2.9	22.4 ± 0.6	2.2	330 ± 50

[1] The uncertainty in the average of the means and ξ is the 99.7% confidence interval derived from the standard error.

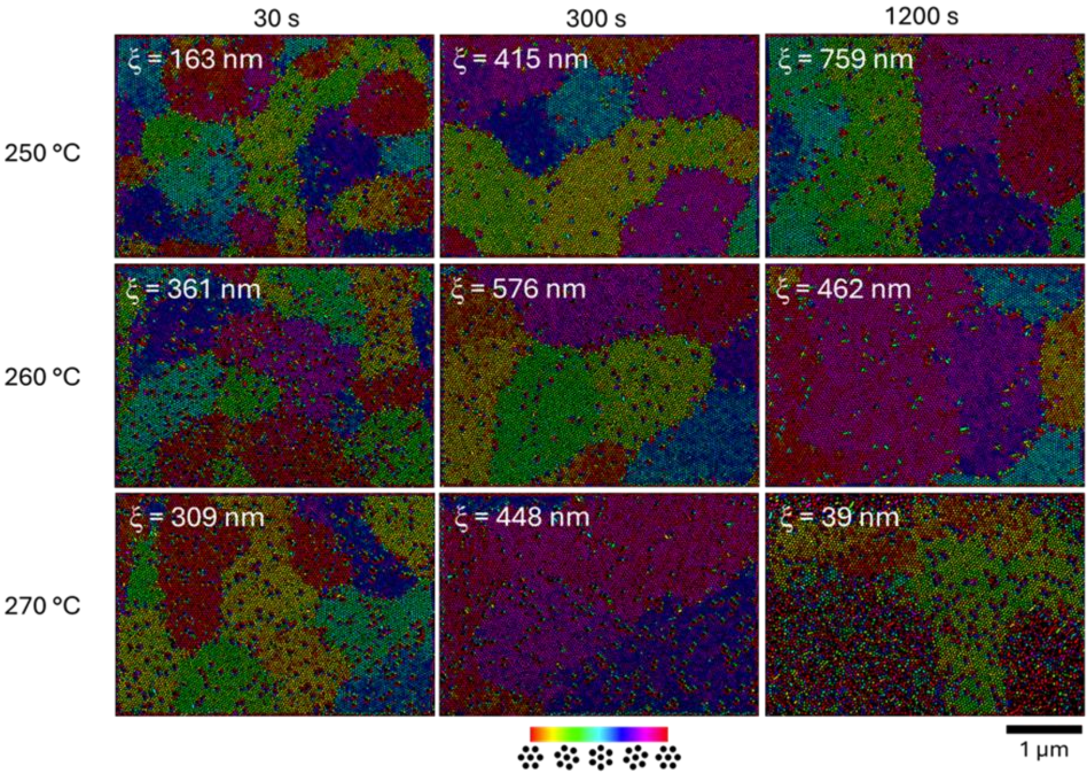

Figure S6: Exemplary orientation maps of self-assembled hexagonal hole patterns computed from low-magnification SEM images of ~50 nm thick films of the PS-b-PMMA/PS/PMMA diblock copolymer/homopolymer blend described in the text. The films were annealed at the given temperatures and for the given times (in seconds) as labelled by row and column, respectively. The polymer films were all ~50 nm thick and were annealed for 5 minutes at 250 °C. Samples were infiltrated and etched as described in the text. The corresponding orientational decay length ξ is listed at the top of each map.

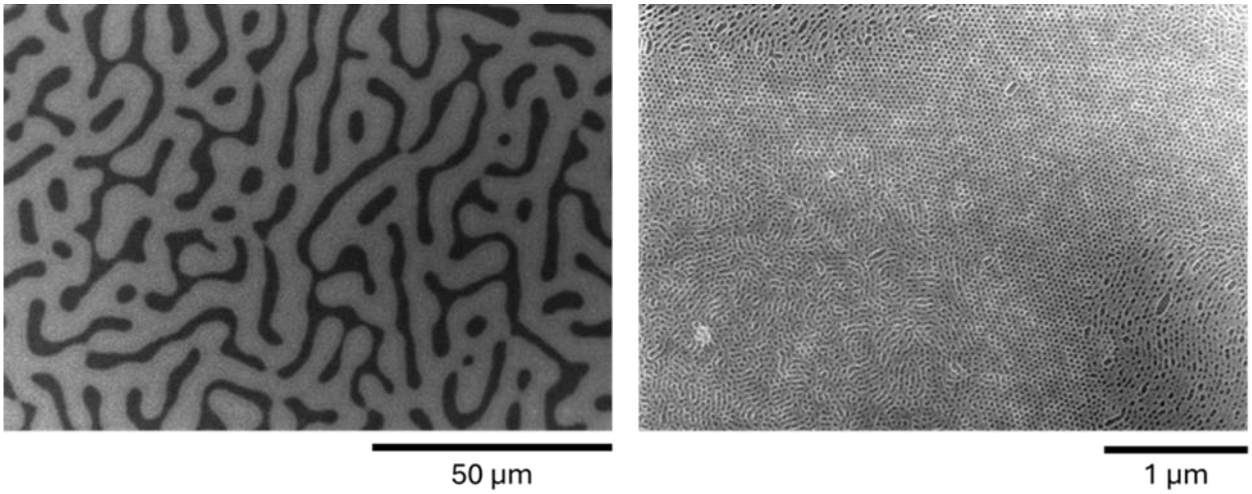

Figure S7: Low (left) and high (right) magnification SEM images showing vertical to horizontal cylinder reorientation in a PMMA/PS/PMMA copolymer/homopolymer blend film annealed for 1200 seconds at 270 °C. The dark regions in the low-magnification SEM image are regions where horizontal cylinders and predominant. Edges of these regions can be seen in the upper left and lower right corners of the high magnification SEM image.

Table S3: Image analysis results for ~50 nm thick PS-b-PMMA/PS/PMMA diblock copolymer/homopolymer blend film samples annealed for 0.5 to 20 minutes at temperatures from 250 to 270 °C.

Annealing Conditions		Period (nm)		Diameter (nm)		ξ (nm)
Temp. (°C)	Time (min)	Mean[1]	Standard Deviation	Mean[1]	Standard Deviation	
250	0.5	38.0 ± 0.1	4.1	22.7 ± 0.1	2.9	164
	5	38.3 ± 0.1	3.2	23.3 ± 0.1	2.5	415
	20	38.5 ± 0.1	3.2	22.8 ± 0.1	2.7	759
260	0.5	37.9 ± 0.1	4.6	21.5 ± 0.1	3.0	361
	5	38.1 ± 0.1	3.3	21.6 ± 0.1	2.5	577
	20	39.1 ± 0.1	3.2	24.0 ± 0.1	2.7	462
270	0.5	37.8 ± 0.1	3.8	21.9± 0.1	2.8	309
	5	38.2 ± 0.1	3.4	21.7 ± 0.1	2.8	448
	20	42.4 ± 0.1	5.0	23.2 ± 0.2	4.5	39

[1]Each time and temperature corresponds to an orientation map presented in Figure S6. The uncertainty in the means represents the 99.7% confidence interval derived from the standard error.

Supplementary note S3: Transport measurement at different V_{SL} potentials

To assess the impact of grain rotation on the remote SL potential, two sets of electronic transport data were measured along the length of the graphene device. The electrical resistivity (ρ_{xx}) was measured between the X_1X_2 and X_2X_3 sensing probes depicted in Fig. 3a, 3b and plotted as a function of the carrier density (n), as shown in Figs. S8a and S8b. These measurements were performed by sweeping the top gate voltage (V_{tg}) while stepping the back gate voltage (i.e. V_{SL}) from 20 V to -42 V.

The graphene device also forms a moiré structure due to the rotational alignment between hBN and graphene. This moiré structure exhibits an additional resistance peak corresponding to a secondary Dirac point at a carrier density of -3.8×10^{12} cm^{-2}. This corresponds to a moiré wavelength of 10.9 nm (see the Brown-Zak analysis in note S6). Notably, the data in Fig. S8 revealed the appearance of additional resistance peaks near this moiré-induced secondary Dirac point when V_{SL} is reduced to form an electrostatic remote SL in graphene. The physical origin of these additional resistance features remains unclear, warranting further dedicated studies.

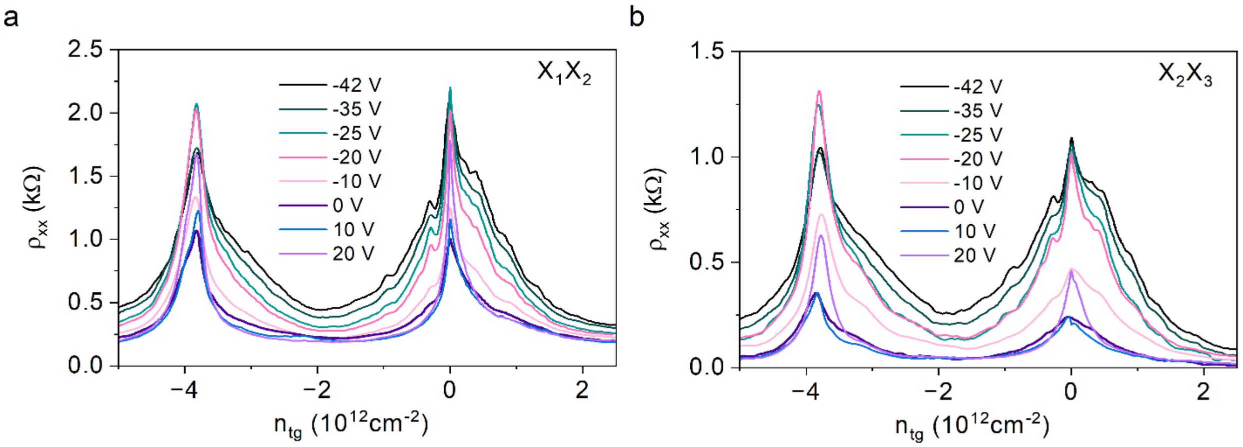

Fig. S8: Electronic transport at zero field. Transport measurement results of the graphene device at different V_{SL} along (a) X_1X_2 and (b) X_2X_3 sensing probes. Notice the presence of a moiré-induced secondary Dirac point, which evolves with reducing V_{SL} by exhibiting additional features.

Supplementary note S4: AlO_x residual charge calculation

The electronic transport measurements show that the SL potential is turned off at V_{SL}=20 V. At this voltage, Δn_{SL}=0, meaning the carrier densities in graphene above both the hole and solid regions of the nanopatterned dielectric are equal.

However, without residual fixed charges in AlO_x, applying V_{SL} = 20 V would create an uneven carrier density between the hole and solid regions of the nanopatterned dielectric (see Fig. S9a). This analysis was performed using the COMSOL model described in Supplementary Note S1. As expected, the calculated carrier density in graphene above the solid AlO_x regions at this V_{SL} is higher than in the hole regions. This suggests that the residual fixed charges in AlO_x are negative, cancelled by the positive V_{SL}. The difference in carrier densities between the solid and hole regions at V_{SL} = 20 V provides a reasonable estimate for the fixed charges in AlO_x, and given by:

$$N_{s,AlOx} = -(\underline{n}_{AlOx} - \underline{n}_{hole}) = -(14.7 \times 10^{11} - 8.8 \times 10^{11}) = -5.9 \times 10^{11} cm^{-2} \quad (1)$$

This calculation can be validated by examining how V_{SL} shifts the charge neutrality point (CNP) voltage in electronic transport measurements. A shift in CNP voltage of graphene indicates electrostatic doping, with a negative V_{CNP} signifying electron doping in graphene. At V_{SL}=20 V, we measured V_{CNP}=-1.45 V. The corresponding electron doping density is estimated as:

$$n_{s,tg} = \frac{C_{tg} \times 1.45\ V}{e} = 8.3 \times 10^{11}\ cm^{-2} \quad (2)$$

The average carrier density above the holes ($\underline{n}_{hole}$), calculated in equation (1), is close to the estimated electron doping density from equation (2). This consistency provides confidence in our approach for estimating the residual fixed charge density in AlO_x.

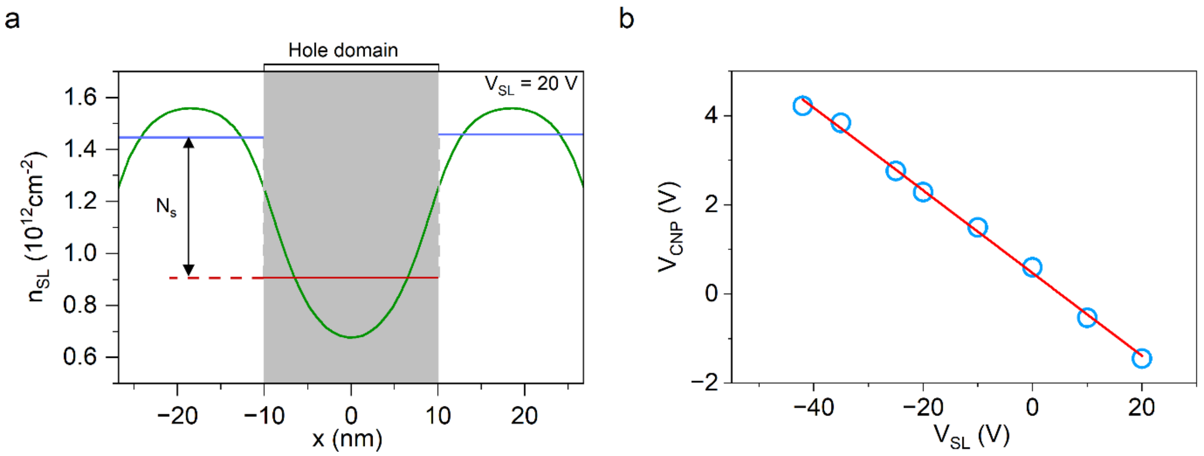

Figure S9: Estimation of fixed charge density in AlO_x. (a) Estimated carrier density in graphene above the solid AlO_x and hole regions of the nanopatterned dielectric at V_{SL}=20 V. The red and blue lines represent the average carrier density in graphene above the hole and solid regions of the nanopatterned dielectric, respectively. (b) The plot of V_{CNP} shifts as a function of applied V_{SL}.

Supplementary note S5: Magnetoresistance measurements

Fig. S10 shows the magnetotransport response of the graphene between X_1X_2 and X_2X_3 sensing probes for the full range of the measured carrier density.

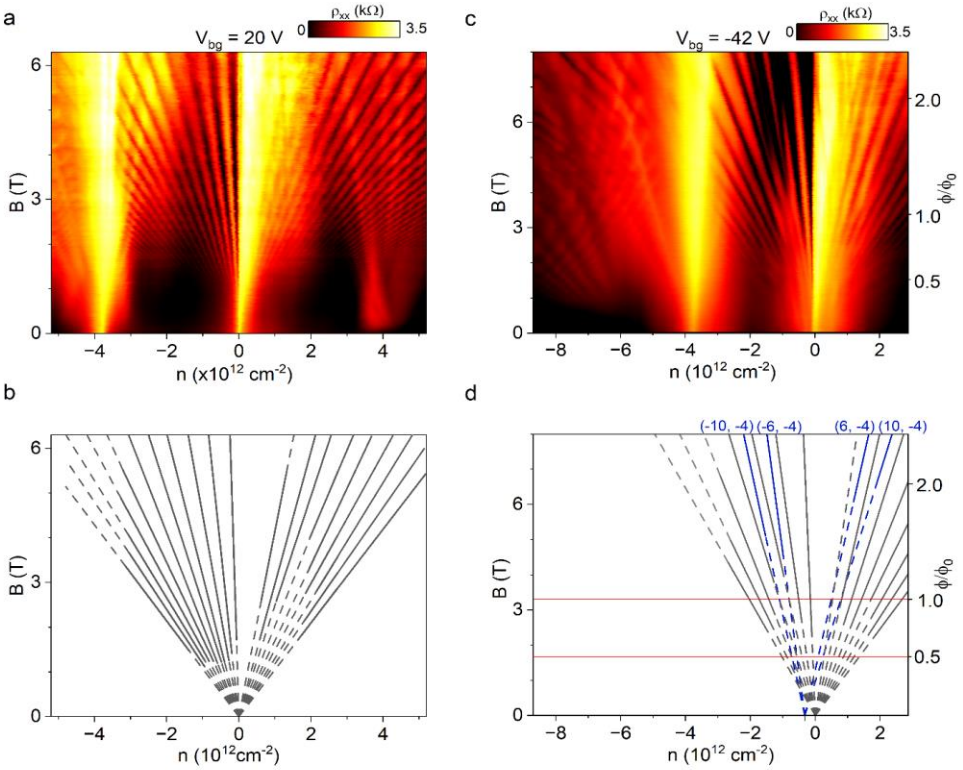

Fig. S10: Full-range magnetoresistance data. The magnetoresistance data of the graphene measured between X_1X_2 sensing probes at (a) V_{SL}=20 V, and (c) V_{SL}=-42 V. The data reveal the presence of quantum Hall states originating from the graphene-hBN moiré structure. The corresponding Wannier diagrams at (b) V_{SL}=20 V, and (d) V_{SL}=-42 V.

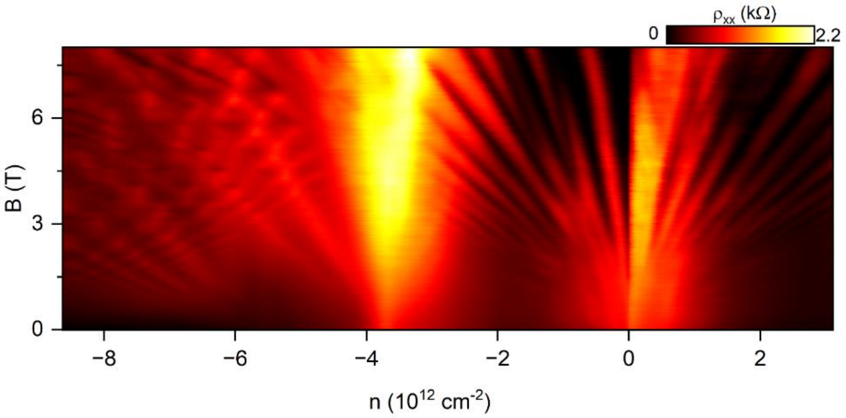

Figure S11: Magnetoresistance measurement between X_2X_3 sensing probe. The data reveal the presence of Hofstadter minigaps, confirming the effectiveness of the templated BCP nanopatterns in inducing electrostatic remote SL across the entire range of the graphene device.

Supplementary note S6: Moiré SL wavelength analysis

We calculated the moiré SL wavelength using the Brown-Zak oscillations. At certain magnetic field values, the fan diagrams originating from both the primary, secondary, and tertiary Dirac points intersect, which is generated by Brown-Zak oscillations. The SL

wavelength can be evaluated from the period of these oscillations, $1/B_0$. From the Brown-Zak oscillations, the moiré wavelength of this graphene device is 10.9 nm, corresponding to $n_s = 9.6\ \times 10^{11}\ cm^{-2}$.

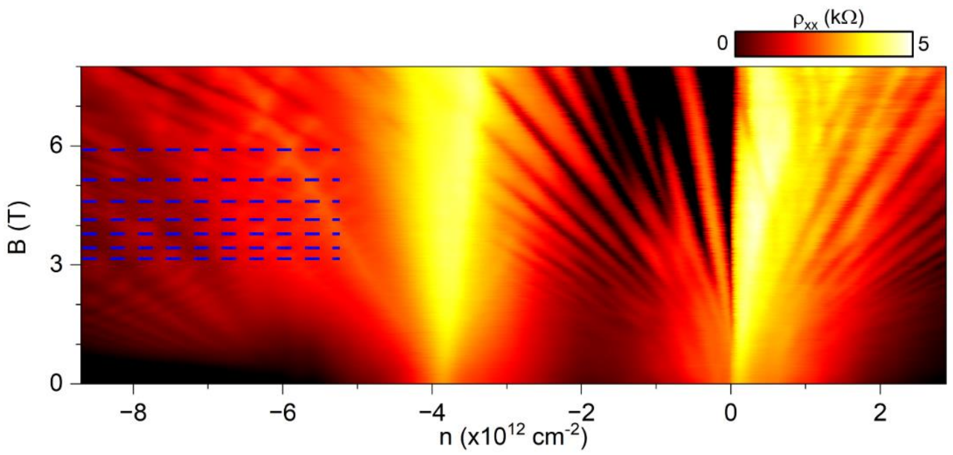

Figure S12: Evaluation of the moiré wavelength. We analyzed the Brown-Zak oscillations for calculating the moiré wavelength of the graphene device in our study.
